# Good enough processing: what have we learned in the 20 years since Ferreira et al. (2002)?

**DOI:** 10.3389/fpsyg.2024.1323700

**Published:** 2024-01-24

**Authors:** Candice Frances

**Affiliations:** Psychology of Language Department, Max Planck Institute for Psycholinguistics, Nijmegen, Netherlands

**Keywords:** good enough processing, language processing, heuristics, depth of processing, ambiguity underspecification

## Abstract

Traditionally, language processing has been thought of in terms of complete processing of the input. In contrast to this, Ferreira and colleagues put forth the idea of good enough processing. The proposal was that during everyday processing, ambiguities remain unresolved, we rely on heuristics instead of full analyses, and we carry out deep processing only if we need to for the task at hand. This idea has gathered substantial traction since its conception. In the current work, I review the papers that have tested the three key claims of good enough processing: ambiguities remain unresolved and underspecified, we use heuristics to parse sentences, and deep processing is only carried out if required by the task. I find mixed evidence for these claims and conclude with an appeal to further refinement of the claims and predictions of the theory.

## 1 Introduction

Traditionally, theories of language comprehension have assumed that we create full, detailed representations of sentences. They claim that people understand sentences much like a linguist would, analyzing them fully and arriving at an unambiguous interpretation. Some researchers have argued that sentences are not fully analyzed incrementally—meaning, bit-by-bit as they are read or heard—, but rather require reanalysis or more than one stage of processing to be fully comprehended (e.g., Frazier and Fodor, 1978). In other words, they propose we carry out multiple (generally, two) run-throughs or stages to analyze a sentence. In the cases in which the signal is ambiguous, they claim that we either activate parallel representations for each possible understanding of the sentence (e.g., constraint satisfaction models; see Trueswell and Tanenhaus, 1994 for a proposal and Frazier 1995 for a critique) or that we reanalyze the sentence to reach an unambiguous representation of it (e.g., Frazier and Fodor, 1978). In other words, theories generally agree that we arrive at an unambiguous understanding of sentences we hear or read (e.g., MacDonald et al., 1994).

Although a complete analysis and unambiguous understanding would perhaps in some ways be ideal, such detailed comprehension may not always occur and can be quite expensive. Good enough processing posits that people regularly engage in superficial analyses of linguistic signals, not expending effort in disambiguating or creating detailed representations of a signal’s grammar and syntax.

### 1.1 What is unique about good enough processing?

In contrast to traditional sentence comprehension theories and keeping in mind the issues mentioned above, Ferreira et al. (2002) proposed that processing is only “good enough” for the task at hand. The authors seem to claim that this processing involves both syntax (speaking of syntactic analysis) and semantics (addressing semantic representations). This approach gives weight to a key issue: detailed analyses are resource-heavy and often unnecessary. Importantly, they advocate for the role of the task, arguing that the depth of syntactic processing depends on the purpose at hand, with comprehension often requiring only enough processing to produce an appropriate and timely response. They presented evidence from misinterpretations of garden-path sentences (e.g., thinking Ana dressed the baby in *While Ana dressed the baby played in the crib*) and passive sentences (e.g., thinking the dog bit the man in *The dog was bitten by the man*) as support for the idea that we do not ordinarily fully analyze sentences. In the first case, participants entertained the interpretation that the baby both was dressed and played, suggesting that the participants did not fully overwrite the initial misinterpretation (i.e., “While Ana dressed the baby” as a unit) and maintained a representation that was not faithful to the signal. In the second case, participants often misinterpreted the dog as the agent of the passive sentence, suggesting that when the syntax is complex and contradicts common sense and superficial analyses, sentences are often misinterpreted. Ferreira and colleagues advocate for what they call good enough processing, namely, somewhat partial or superficial processing that is ordinarily done when one is listening or reading information. This type of processing allows for interpretations not licensed by the grammar and so we use non-syntactic information to parse sentences—e.g., plausibility, semantic relatedness, and heuristics. This information takes advantage of prior knowledge about the world and language to simplify or speed up processing.

At first glance, the idea of good enough processing is quite appealing. Intuitively, economizing on processing makes a lot of sense and aligns with our knowledge of cognitive processes. Why would we carry out costly processes, some of which may even require explicit effort, if they are not required? The notion that we do not naturally, automatically, and mandatorily run linguist-level analyses on the language we hear or read fits well with our common sense intuitions and observations. If we constantly carried out deep processing, conversations would be extremely cumbersome but also an interaction at a grocery store would always be remembered as well as an academic lecture. Looking at how quickly we respond in conversation—often overlapping turns—with no obvious comprehension issues or, conversely, observing how often we misunderstand unambiguous statements, it is difficult to deny that we must be carrying out some kind of processing shortcut (see Federmeier, 2007 and Pickering and Garrod, 2007 for explanations using predictive processing). From a research perspective, this idea of good enough processing is extremely appealing in that it helps explain a wide variety of often surprising results such as errors in comprehending unambiguous sentences (Ferreira, 2003) or lingering misinterpretations (Patson et al., 2009).

The idea of good enough processing provides a nexus between what linguists study about language and how it is actually processed by the individual. For people engaging in daily language use, the full parsing and understanding of a sentence might not always be the goal. This theory takes goals into consideration and allows for the possibility that people interpret only to the extent that they need to. The idea of good enough processing also takes into consideration that the individual has prior knowledge of how language works and tries to economize resources (see Federmeier, 2007 and Pickering and Garrod, 2007 for similar claims in terms of predictive processing). In other words, if some shortcuts or heuristics spare resources, it is reasonable to believe people would employ them.

### 1.2 The current review

In the current paper, I will formalize these claims put forth in Ferreira et al. (2002) and then evaluate the evidence from papers testing these claims directly in the 20 years since that original publication. The focus of the current work is to assess the extent to which these claims have been tested and supported or challenged. According to Scopus, as of November 1^st^ 2022 there were 531 works citing Ferreira et al. (2002). Most of these citations either took the ideas of good enough processing as background information for their studies or as partial explanations for their results. A few of them addressed the claims of this theory more directly.

I have summarized their claims into the following three points:

- Ambiguities remain unresolved and underspecified.
- We use heuristics to parse sentences instead of relying on a full syntactic analysis.
- Deep processing is only carried out if required by the task.

It should be noted that the ideas of good enough processing have been extended to production (Goldberg and Ferreira, 2022) and there is some support for the idea that language users may produce suboptimal messages based on word availability (e.g., Koranda et al., 2022). Nevertheless, the focus of this paper will be on comprehension as put forth in Ferreira et al. (2002) and subsequent papers.

## 2 Methods

I assessed the direct evidence for these three claims that have arisen from the seminal 2002 article. More specifically, the present study collated and reviewed all the empirical studies that test the claims of good enough comprehension published between 2002 and 2022. I downloaded the list of 531 works citing Ferreira et al. (2002) from Scopus on November 1st, 2022 and took this as my starting point. The focus of this review is healthy adult populations. It should be noted that there may be more relevant papers often predating Ferreira et al. (2002) or not citing this paper. These will systematically not be included here.

I removed non-empirical studies: books, book chapters, and reviews (146). Then, I removed the articles that did not directly test the claims put forth here because they either only cited Ferreira et al. (2002) in the introduction (244) or only in the discussion (208), leaving 81 where the study was cited in both sections. The vast majority of articles that cited good enough processing in the introduction mentioned the idea in passing. Most of the articles that cited this paper in the discussion section did so as a *post hoc* explanation of their results or as a possible explanation for unexpected results. I considered citing the paper in both sections a necessary condition if they were to test the claims of good enough processing. Finally, of the 81 articles citing the 2002 paper in both the introduction and discussion, I removed all studies on children and patients (60), leaving 21 that tested one or more of these three claims directly on healthy adult populations. I then established which of the three initial claims they addressed and will report them in groups by claim.

## 3 Summary of papers by claim

### 3.1 Ambiguities remain unresolved and underspecified

The good enough processing approach claims that ambiguities in language remain unresolved or underspecified. The 11 studies described below used varied approaches to test this idea such as garden-path sentences, models of processing, and processing of repairs. In the following paragraphs, I will summarize each of these studies and provide general conclusions.

Gilbert et al. (2021) assessed the claim that readers only determine the meaning of ambiguous words when required by the task, otherwise maintaining ambiguity. They operationally defined ambiguous words as polysemous words (e.g., bank as in either money bank or river bank) that could temporarily be ambiguous. They studied the lexical-semantic retuning of polysemous words—i.e., the “redefinition” of the word after being disambiguated by the context. They tested whether this occurs immediately upon hearing the ambiguous word (requiring prior disambiguation in order to access the correct meaning) or if it can happen later depending on the level of reinterpretation needed (benefitting from subsequent disambiguation). In the first case, lacking prior disambiguation, the most frequent meaning of the word would be taken and would not benefit from later disambiguation. In the second case, the meaning would remain ambiguous until the participant needs to access the meaning of the word, benefiting from later disambiguation. They used a priming paradigm in which native English speakers encountered the less frequent meaning of ambiguous (polysemous) words (e.g., *bank* as in river bank is less frequent than *bank* as a money bank) in priming sentences that featured the ambiguous word with its low-frequency use. They tested whether this primed the low-frequency interpretation in subsequent meaning preference tasks in which participants heard the word and produced the first associated word that came to mind. They tested whether these priming effects depended on the initial encounter being immediately unambiguous (i.e., the disambiguating context occurring before the word) or if a reanalysis of the word’s meaning was sufficient (i.e., the disambiguating context occurring after the word).

Example stimuli.

Pre-word disambiguation: *The old man had a long way to swim as he headed for the **bank**.*

Post-word disambiguation: *The old man headed for the **bank** but he had a long way to swim*.

Additionally, they manipulated the task that came after reading to require either shallow (reading for comprehension) or deeper processing (assessing whether a probe word was related to the sentence). The association tasks were carried out after the reading was done. They found consistent priming for sentences in which word-meaning disambiguation occurred before the word, but inconsistent effects when this occurred after. In the latter case, the type of processing required by the task affected the results, with priming occurring only when deeper processing was required. This provides evidence that the meaning of the ambiguous word remains underspecified and is not reassessed unless required by the task, supporting Ferreira et al.’s (2002) general claim that ambiguities remain unresolved.

Swets et al. (2008) and Tan and Foltz (2020) looked at underspecifying ambiguity from a syntactic point of view. Both studies tested whether, on the one hand, readers construct alternative structures or reanalyze ambiguous attachments or, on the other, strategically underspecify interpretations. Participants—native English speakers in the first case and Chinese learners of English in the second—read syntactically ambiguous sentences (such as *The maid of the princess who scratched herself in public was terribly humiliated*). These contained relative clauses (*who scratched herself in public*) that could attach to different noun phrases (either *the maid* or *the princess*). These were contrasted with disambiguated sentences with attachments to the first (N1) or second noun (N2).

Ambiguous: *The maid of the princess who scratched herself in public was terribly humiliated.*

N1 Attachment: *The son of the princess who scratched himself in public was terribly humiliated*.

N2 Attachment: *The son of the princess who scratched herself in public was terribly humiliated*.

If sentences were being processed algorithmically—requiring later reanalysis to “fix” the error—as more traditional perspectives would suggest, ambiguous sentences should take longer to read and process than disambiguated ones, simply because they are more difficult to parse. Nevertheless, Swets et al. (2008) and Tan and Foltz (2020) found that self-paced reading was faster for ambiguous sentences than for non-ambiguous ones, suggesting that participants did not try to disambiguate them. They interpreted this as support for the claim that syntactic ambiguities remain unresolved using self-paced reading and offline questions. This aligns with the results originally cited by Ferreira et al. (2002) from Traxler et al. (1998) suggesting that rather than attempting to decide on a specific parsing, participants simply maintained the ambiguity.

Logačev and Vasishth (2016) tested the fit of different underspecification models. They used computational modeling and compared the fit of partial specification and non-specification models reanalyzing the data from Swets et al. (2008) described above. The difference between the two tested models is that while the first assumes the storage of information about potential attachment sites, the latter does not. In other words, the first adheres to the idea of unresolved ambiguity, whereas the latter suggests participants are forced to guess at the time of answering the probe question. They found that while the non-specification models fit the data better, partial-specification models affected the answer choice in 17% of trials. The authors conclude that, if underspecification occurs and explains the ambiguity advantage, it is rare and does not explain the majority of incorrect responses, providing partial evidence for good enough processing.

Nakamura and Arai (2016) tested the underspecification of syntactic structures. They tested whether, in garden-path sentences, readers preserve the initial incorrect analysis following structural reanalysis even when the initial misanalysis is not pragmatically plausible. They argued that a possible confusion in traditional studies on garden-path sentences is that they leave the object of the first portion of the sentence implicit—e.g., *herself* is implicit in *while Anna dressed.* This allows for the interpretation that the initial analysis, although not syntactically licensed, might still be true or pragmatically inferred. They tested Japanese participants using self-paced reading of garden-path sentences and offline questions. In their study, they specifically used garden-path sentences in Japanese that did not allow for this inference. These garden-path sentences had the traditional ambiguous region with possible main or relative clause attachment but contained either main clause biased, relative clause biased, or neutral nouns.

Main clause biased noun: *The baby stared at the actress who spilled the milk.*

Relative clause biased noun: *The baby stared at the actress who spilled the champagne.*

Neutral noun: *The baby stared at the actress who spilled the drink*.

They found that the initial misanalysis persisted even in cases where no pragmatic inference could be made. They interpreted this as participants being unable to fully erase the initial incorrect analysis. This suggests that participants maintain incompatible representations without fully disambiguating between them, supporting the idea that we maintain ambiguity, as proposed by good enough processing.

Von Der Malsburg and Vasishth (2013) also studied syntactic underspecification. They tested specifically whether readers underspecify attachment. The authors compared the attachment decisions of high- and low-memory-capacity readers in garden path sentences. Participants read Spanish garden-path sentences such as in (1) that had an ambiguous or unambiguous attachment of the adverbial clause, while their eye movements were tracked.

(1) El profesor dijo que los alumnos se levantaran del asiento…

[The teacher said that the students had to stand up from their seats …].

High attachment: [AdvC] cuando los directores **entraron** en la clase de música.

[when the directors came into the music class].

Low attachment: [AdvC] cuando los directores **entraran** en la clase de música.

[when the directors come into the music class].

No ambiguity: [AdvC] si los directores entraban en la clase de música.

[if the directors come into the music class].

The different possible semantic interpretations varied in terms of the temporal order of events, which participants were asked about after every sentence. The authors ran a scanpath analysis on the eye-tracking data. They found that the pre-verbal region was read faster when it was ambiguous and this effect was larger in readers with a low working memory score. They found that readers with high memory capacity commit to attachment more often—or, in other words, underspecify less. This, in turn, leads to more errors and a greater need for reanalysis to recover from garden-pathing. The authors interpreted these results in support of the idea of underspecification, aligning with the proposal by Ferreira et al. (2002).

Chromy (2022) tested underspecification in garden-path sentences. He tested whether readers have differentially higher errors on comprehension questions targeting the initial misanalysis compared to questions targeting an analysis that is not syntactically licensed at any point. He tested native Czech speakers on their comprehension of garden-path sentences. The stimuli were composed of two coordinated sentences joined with *and* that led to a temporary misanalysis: two objects in the first clause joined by *and* rather than each object corresponding to a different clause. In other words, the first phrase was presented in canonical order (*Boys chased a dog*) and the second phrase was presented in an OS order for the garden path sentence (*a cat-ACC in attic worried grey rodents-NOM,* meaning *“grey rodents in the attic worried a cat”*) and the canonical SO order for the non-garden path version (*grey rodents-NOM in attic worried a cat-ACC,* with the same meaning).

Garden-path condition.

Kluci honili psa a kočk-u v podkroví.

[Boy-NOM.M.PL chase-3PL.M.PST dog-ACC.M.SG and cat-ACC.F.SG in attic-LOC.N.SG.

znepokojovali šediví hlodavci.

worry-3PL.M.PST grey-NOM.M.PL rodents-NOM.M.PL].

[Boys chased a dog and grey rodents in the attic worried a cat.]

Non-garden-path condition.

Kluci honili psa a kočk-a v podkroví.

[Boy-NOM.M.PL chase-3PL.M.PST dog-ACC.M.SG and cat-NOM.F.SG in attic-LOC.N.SG.

znepokojovala šedivé hlodavce.

worry-3SG.F.PST grey-ACC.M.PL rodents-ACC.M.PL].

[Boys chased a dog and a cat in the attic worried grey rodents.]

The author looked at reading times and answers to comprehension questions targeting the initial misanalysis as well as misinterpretations unrelated to possible parses of the sentence. He replicated the finding that comprehension questions targeting the initial misanalysis yield significantly higher rates of incorrect answers after garden-path sentences, but this increase also extended to the misinterpretations unrelated to possible parses. The author suggests that rather than the initial misanalyses interfering with the correct analysis, garden-path sentences are just difficult to parse, leading to higher errors overall. I see an alternative interpretation of this. Grammatical sentences with only one correct syntactical analysis are sometimes treated as ambiguous (Keshev and Meltzer-Asscher, 2021). In these kinds of complex sentences, participants will opt for ungrammatical analyses in their interpretation. From our perspective, this could also be viewed as a general ambiguity in comprehension of complex sentences leading to the acceptance of several interpretations—even if not licensed by the grammar or unrelated to the ambiguous portion of the sentence. From that perspective, this still shows evidence for ambiguity, just not for the persistence of the initial misanalysis.

Qian et al. (2018) tested ambiguity as the cause of misinterpretation of garden-path sentences. This study addresses the likelihood of an incomplete reanalysis as the cause of the misinterpretation of garden-path sentences. They tested native English speakers on optionally transitive (verbs that do not require a direct object, but accept one, such as *eat* or *clean*) and reflexive absolute transitive verbs (verbs that, when no direct object is given are considered reflexive, such as *dress* or *bathe*) in ambiguous and unambiguous sentences.

Optionally transitive verb ambiguous condition:

*While the man hunted the deer that was brown and graceful ran into the woods*.

Reflexive absolute transitive verb ambiguous condition:

*While Anna dressed the baby who was cute and small spit up on the bed.*

Optionally transitive verb unambiguous condition:

*While the man hunted, the deer that was brown and graceful ran into the woods*.

Reflexive absolute transitive verb unambiguous condition:

*While Anna dressed, the baby who was cute and small spit up on the bed.*

Sentences were disambiguated for the unambiguous condition by adding a comma after the first verb. The authors used self-paced reading and ERPs online as well as offline comprehension questions. They hypothesized that correct answers to comprehension questions in the ambiguous condition were due to a complete reanalysis and posit that this reanalysis should be reflected in increased reading times in the disambiguation region. Therefore, they compared reading times for trials with correct and incorrect answers. Their results showed no increase in reading times for correct compared to incorrect trials. Furthermore, they used P600 effects (a positive going electrophysiological signal at around 600 ms post-stimulus) on the disambiguating region as an index for the effort put into reanalysis in that region. The expectation was for a larger effect to be present when comprehension questions were answered correctly rather than incorrectly. They found evidence for reanalysis in the disambiguating region: a P600 effect with a larger waveform for the ambiguous versus unambiguous condition. In contrast, the size of the effect was not related to whether the question was answered correctly or not, following the reading time results. This shows that incorrect answers were not due to reduced effort or lack of reanalysis. The authors interpreted this as evidence against good enough processing. Nevertheless and in contrast to the online results, they found that likelihood ratings for the garden path sentences predicted accuracy well. In other words, whether the incorrect interpretation was plausible and to what extent, rather than whether it was syntactically licensed predicted whether people responded to comprehension questions correctly. We can interpret this as evidence in favor of the use of heuristics—such as the likelihood of the event in the misinterpretation—as the cause of misinterpretation, supporting this idea put forth by good enough processing.

Slattery et al. (2013) tested ambiguity in incomplete reanalysis. They tested two possibilities in dealing with the types of ambiguities present in garden-path sentences. First, the traditional view in that at the critical point in the sentence, the ambiguity either is detected and reanalysis occurs or is not noticed and the initial misparse is maintained. The second option, following the good enough view, is that processing is incomplete even before reaching the ambiguity and remains approximate, allowing for the misinterpretation to linger. They used eye tracking to test native English speakers on reflexive binding and gender mismatch in garden path sentences.

Garden path match sentence:

*After the bank manager telephoned David’s father grew worried and gave **himself** approximately five days to reply*.

Garden path mismatch sentence:

*After the bank manager telephoned David’s mother grew worried and gave **himself** approximately five days to reply*.

Non-garden path match sentence:

*After the bank manager telephoned, David’s father grew worried and gave **himself** approximately five days to reply*.

Non-garden path mismatch sentence:

*After the bank manager telephoned, David’s mother grew worried and gave **himself** approximately five days to reply*.

The gender manipulation relied on changing the gender of the subject—i.e., replacing *father* with *mother* in the example. The non-garden path version included a comma after the first verb (“telephoned,” in the example). They found a slowing down in the disambiguation region (i.e., the region of the second verb, *grew* in the example above) as well as a gender mismatch effect (i.e., slower reading times in the reflexive and end-of-sentence regions for the conditions in which there was a gender mismatch—in the example above, the mismatch condition replaced *father* with *mother*). They interpreted this as evidence that participants created a detailed representation of the syntactic structure of the sentence as the parser attempted to link the reflexive pronoun to its antecedent. In a second experiment, they used two-sentence texts to study the processing and structures built from garden-path sentences in terms of spillover effects to the second sentence as well as plausibility effects of the incorrect parse.

Non-Garden Path/Plausible.

*While Frank dried off, the truck that was dark green was peed on by a stray dog. Frank quickly finished drying himself off then yelled out the window at the dog.*

Garden Path/Plausible.

*While Frank dried off the truck that was dark green was peed on by a stray dog. Frank quickly finished drying himself off then yelled out the window at the dog.*

Non-Garden Path/Implausible.

*While Frank dried off, the grass that was dark green was peed on by a stray dog. Frank quickly finished drying himself off then yelled out the window at the dog.*

Garden Path/Implausible.

*While Frank dried off the grass that was dark green was peed on by a stray dog. Frank quickly finished drying himself off then yelled out the window at the dog.*

In this experiment, they found lingering effects of the misparse in the next sentence, evidenced by a slowdown in the reading of a critical region in the following sentence (consistent only with the correct interpretation of the garden-path sentence) when the garden path was plausible. In sum, although a proper structure is built following the first reading of the sentence (Experiment 1), the remnants of earlier attempts to parse the sentence linger and affect offline responses (Experiment 2). The authors interpreted these results in support of good enough processing.

Frazier and Clifton (2015) tested the underspecification of syntactic blends and double-quantifier sentences. They tested whether native English-speaking participants repair utterances in which the speaker’s intention does not align with the grammatically-licensed compositional interpretation of the signal. They had native English speakers listen to or read sentences and judge the acceptability of blends and double quantifier sentences.

Syntactic blend: *A passerby rescued a child from almost being run over by a bus*.

Double quantifier: ***Many** students **often** turn in their assignments late*.

Theoretically, syntactic blends arise from a blending of expressions. In the example above, “rescued from fatality” and “there was almost a fatality” blend. Double quantifier sentences simply have two quantifiers for the same action. The authors looked at responses in which participants incorrectly marked double quantifier sentences as acceptable. They found slower reading times for incorrectly accepted sentences. The authors suggest that instead of underspecification, the same observations can be explained by participants using their knowledge of common or likely errors and deducing the intention of the person emitting the message. Although the authors argue against good enough processing, their conclusion that participants are using their knowledge or heuristics aligns with the good enough processing account. It is also worth noting, that there was no slowdown or difference in reading times for blended sentences that were incorrectly accepted, only in the double quantifier case, further weakening the interpretation against good enough processing.

Paape et al. (2020) tested whether ambiguities remain unresolved when processing written sentences. Paape and colleagues studied so-called depth charge sentences (e.g., *No head injury is too trivial to be ignored*). In a series of five experiments, they tested German speakers using self-paced reading and eye-tracking online as well as sensibleness and grammaticality ratings and sentence completion offline in two experiments. Their goal was to establish whether the misunderstanding of these sentences (for instance, understanding the above example as “all head injuries must be taken seriously”) was due to a memory overload or ambiguity in the processing of the sentence. When asked to interpret these sentences participants often arrived at a meaning that was sensible but not licensed by the grammar. They found evidence that working memory capacity (measured using an operation span task) had no effect on the illusion, but that world knowledge did (calculated using a linear mixed effects model combining a measure of approval or agreement with the sentence and one of ease of understanding). The authors suggest that rather than experiencing a memory overload, participants run out of motivation and use a shallow, good enough processing strategy. They interpret the results as showing a likely influence of heuristics on the analysis of the sentence, which supports Ferreira et al.’s (2002) claim.

Finally, Schlesewsky and Bornkessel (2006) tested the presence of incomplete reanalysis and ambiguity. They tested native German speakers using word-by-word reading with EEG followed by comprehension questions. They manipulated whether verbs assigned the accusative or dative case and whether the subject was presented before or after the object. All sentences started the same way with a setup (e.g., *Yesterday, someone said that…*) and two case ambiguous nouns, one singular (e.g., *Richard*) and one plural (e.g., *artists*). These were followed by either an accusative (e.g., *sehen*, meaning to see) or a dative (e.g., *danken,* meaning to thank) verb in either singular (placing the first noun as the subject and leading to a subject-object structure) or plural (placing the second noun as the subject, marking an object-subject order). In a second experiment, they used case-marked nouns for the second noun.

Common matrix clause: Gestern wurde erzählt,…

[yesterday was told…]

[‘Yesterday, someone said…’].

A–SO: … dass Richard Künstlerinnen gesehen hat, obwohl…

[… that RichardAMB.SG artistsAMB.PL seen-ACC hasSG although…]

[‘… that Richard saw artists, although…’].

A–OS … dass Richard Künstlerinnen gesehen haben, obwohl …

[... that RichardAMB.SG artistsAMB.PL seen-ACC havePL although…]

[‘… that artists saw Richard, although...’]

D–SO … dass Richard Künstlerinnen gedankt hat, obwohl …

[… that RichardAMB.SG artistsAMB.PL thanked-DAT hasSG although…]

[‘… that Richard thanked artists, although…’].

D–OS … dass Richard Künstlerinnen gedankt haben, obwohl…

[… that RichardAMB.SG artistsAMB.PL thanked-DAT havePL although…]

[‘… that artists thanked Richard, although…’].

They carried out these two ERP experiments testing the N400 and P600 as markers for reanalysis in dative-active verbs and dative-nominative constructions. From their P600 results, they found that dative-active sentences benefitted from the fact that dative-nominative is available as a possible unmarked word order in German, and therefore avoiding full structural reanalysis (leading to smaller P600s)—as explained by a good enough representation. But, on the other hand, they found that their N400 effects, which were unaffected by sentence context, could not be explained by a good enough effect. They conclude that good enough processing explains the reduction in P600 when the unmarked word order is used, but it does not address the invariable presence of N400 effects. Thus, they provide evidence for good enough processing in some cases, but not in others.

#### 3.1.1 Interim discussion

All in all, the studies used different paradigms seeking online and offline evidence for the resolution of ambiguity. They combine several different methodologies—eye-tracking, self-paced reading, ERPs, and offline questions—as well as studying different kinds of phenomena—garden-path sentences, reflexive binding and gender mismatch, syntactic blends and double quantifier sentences, and syntactic and lexical ambiguities. They also use different languages—English, Czech, Spanish, Japanese, and German.

The results are mixed. The online data show that ambiguous sentences—and, in particular, ambiguous regions—are read faster. In contrast, double quantifier sentences that were incorrectly accepted were also read slowly, unlike garden path sentences which show no difference between reading times in correct and incorrect trials. There is an effect of reanalysis in the disambiguation region for garden-path sentences (P600), slowing down in the disambiguation in garden-path sentences, and gender mismatch effect in real-time, all showing no evidence of reduced effort or lack of reanalysis. Nevertheless, there are “hangover” effects of garden-path sentences and the remnants of earlier parses affecting offline responses, and rather than showing memory overload, in some cases, participants run out of motivation and use shallow processing. Additionally, dative-active sentences benefit from being a possible unmarked word order in German and therefore avoiding full structural reanalysis (leading to smaller P600s) but this does not explain N400 effects. The off-line data show that reanalysis does not occur unless required by the task and initial misanalyses persist even when no pragmatic inference can be made, but garden-path sentences are difficult to parse overall rather than showing incomplete reanalysis, and underspecification only explains a small percentage of errors.

It is clear that ambiguities do not remain unresolved in all cases and the specifics of when and why remain somewhat unclear. Nevertheless, the evidence remains in support of the idea that there are situations in which some ambiguities remain unresolved. Furthermore, there is even a suggestion that it is beneficial to do this as it saves memory and reduces the effort required in “fixing” errors when the wrong conclusion is reached. In sum, although they do not provide a conclusive universal answer, these studies do provide evidence that there are cases in which ambiguities do remain unresolved.

### 3.2 We make use of heuristics to parse sentences

A second claim in good-enough processing is that we make use of heuristics to comprehend sentences. These heuristics are simple rules that apply to the majority of cases, allowing a fast and frugal solution that does not rely on a systematic analysis of the specific sentence. These provide schemas that guide the analysis of the sentence in a non-compositional way. In other words, rather than analyzing the structure bit-by-bit, heuristics provide “shortcuts” that speak to the overall structure, rather than calculating it in a bottom-up, additive way. Although this is presented implicitly in Ferreira et al. (2002), this claim is made more explicit in later elaborations [from Ferreira and Patson (2007) on]. The idea here is complementary to the others in that it hypothesizes that rules of thumb provide shortcuts to understanding sentences, saving us the effort of a full, detailed analysis of the sentence. In the following paragraphs, I summarize the five studies that address this claim directly.

Ferreira (2003) tested the use of heuristics when interpreting auditory sentences. In particular, she tested whether using a noun-verb-noun or NVN strategy—a heuristic that says that the first noun in a sentence is the agent and the second is the patient—would explain the difficulty in understanding passives and object clefts. She had participants listen to complex, but unambiguous sentences and had them answer the following comprehension questions orally: who was the agent, who was the patient or theme, what was the action, the location, the color, and when it took place. She compared the comprehension of actives, passives (e.g., The man was bitten by the dog), and object-clefts (e.g., It was the man the dog bit). She found that the sentences that violate the premise of the NVN heuristic were more difficult to understand. In other words, if the first noun in the sentence was not the agent, the sentence became more challenging to parse. In both passive sentences—in which the subject, although it appears first, is the patient—and object clefts, the patient appears before the agent. To test the possibility that the effects were simply due to the frequency of encountering the syntactic structure, she compared the results with subject clefts. These types of structures are infrequent but respect the premise of the NVN strategy. She found that they were understood correctly just as often as canonical active sentences. Importantly, implausible sentences in passive or object-cleft forms were particularly difficult to assign thematic roles to, suggesting an appeal to world knowledge to help parse sentences that violate the NVN heuristic. She concluded that these results support the good enough processing claim that comprehension utilizes some basic heuristics—such as the NVN strategy—and world knowledge to parse syntactically difficult sentences.

Bader and Meng (2018) tested morphological case as a heuristic strategy. Using analogous stimuli and design to Ferreira (2003), they tested native German speakers on active sentences with subject-object or object-subject order—differentiated using case marking—and passive sentences.

Active sentences with subject-object: *Der Koch hat den Braten ruiniert.*

[*The chef_NOM_ ruined the roast_ACC_*].

Active sentences with object-subject order: *Den Braten hat der Koch ruiniert.*

*[The roast_ACC_ ruined the chef_NOM_]*

*[“The roast, the chef ruined.”]*

Passive sentences: *Der Braten wurde vom Koch ruiniert.*

*[The roast_NOM_ was ruined by the chef_ACC_].*

Participants listened to sentences and responded to two kinds of questions: actor/undergoer questions analogous to Ferreira (2003) and plausibility questions. The first requires a post-interpretation and retrieval of the sentence (as it depends on the probe) whereas the second relies on the immediate interpretation of the sentence. Using this paradigm, they tested the use of morphological case in the interpretation of passive sentences and sentences in non-canonical order. They found that participants did not use morphological case marking consistently to answer questions. Participants produced errors only in the first task—asking about roles in the sentence—but not in the second—asking about plausibility. This is especially surprising considering that in order to answer the question about plausibility, thematic roles had to be assigned correctly. The authors interpreted this as evidence against the use of heuristics and in favor of algorithmic processing. Alternatively, this study suggests that morphological case marking—at least in some cases—is not used by participants as a heuristic when answering comprehension questions or processing sentences. In other words, this suggests that morphological case marking may not be one of the heuristics people use, rather than providing evidence against the use of heuristics in general—the good enough processing claim tested here.

Keshev and Meltzer-Asscher (2021) tested the use of heuristics in the form of knowledge of the distribution of structures in a language. They studied the processing of subject and object-relative clauses in Hebrew. Four experiments were carried out using self-paced reading with yes/no comprehension questions and a fifth used rapid visual serial presentation and a sentence completion task. In Experiment 1, participants read sentences with relative clauses that could be subject-relative or a rare kind of object-relative clauses with a post-verbal subject. These two options could sometimes be disambiguated at the verb (in the case of a mismatch between the object and the verb) or the post-verbal subject. Participants showed increased processing costs—slow-down in reading—at the post-verbal subject in both cases, suggesting that they had ignored the grammatical error in favor of the more common subject-relative clause. In a second experiment, they used a more common form of an object-relative clause—namely, an impersonal null-subject clause. In this case, readers no longer mistakenly read the sentence as containing a subject-relative clause. These results were further backed up with the sentence completion task: participants completed sentences in a grammatically incorrect way in order to avoid the rare object-relative clause with a post-verbal subject, but correctly completed the object-relative clause when the more common impersonal null-subject clause was available. The authors argue against a good enough processing interpretation of the results, interpreting the predictions of this view in terms of memory fallibility—i.e., errors are due to incorrect interpretations arising at the moment of reevaluating and answering questions. They argue that good enough processing would predict equal amounts of errors between conditions and does not account for the difference between the rates of the subject relative interpretations depending on the available object relative alternative. They claim that the predictions of good enough processing are based on misparses resurfacing in comprehension offline due to difficulties in inhibiting them. Nevertheless, these results could be interpreted as participants treating unambiguous sentences as ambiguous and that the analysis of the syntactic structure is influenced by the probability of encountering one or another structure—a syntactic likelihood heuristic. Higher likelihood structures are given precedence over lower likelihood structures, interpreting the sentence in a shallow way that allows for minor errors in support of the more likely structure. This way, this study would provide evidence for the use of heuristics—Ferreira et al.’s (2002) second claim.

Dwivedi (2013) tested the use of heuristic strategies (namely, the use of lexical-pragmatic associations of words) preceding algorithmic strategies. She used self-paced reading to assess native English speakers’ interpretations of sentences with quantifier scope ambiguity. She used statements such as *Every boy climbed a tree* which could refer to multiple boys climbing multiple trees (plural interpretation) or all of them climbing the same one (singular interpretation). These were contrasted with unambiguous sentences. This first sentence was followed by a disambiguating sentence.

Ambiguous sentence: *Every boy climbed a tree.*

Unambiguous sentences: *Every boy climbed that tree/those trees.*

Disambiguating singular sentence: *The tree was in the park.*

Disambiguating plural sentence: *The trees were in the park*.

She also included either a lexical-pragmatic bias (whether there is a preference for the plural interpretation) or increased task demands (either answering a number question or no question). She found that participants read the ambiguous nouns faster than unambiguous ones, suggesting these ambiguities remained unresolved and analyzed in a shallow manner. Even in the case of heavily biased sentences, there was no evidence of disambiguation in the continuation sentence—meaning the “misaligned” continuation was read just as fast as the aligned one. When asked questions, participants took longer to read the dispreferred single continuations regardless of ambiguity, suggesting task-dependent deep processing. She interpreted this as participants using lexical-pragmatic biases as a heuristic informing number interpretation. These three conclusions—the use of shallow processing, task-dependent processing depth, and the use of heuristics—were all interpreted in support of good enough processing.

Additionally, in their study on the resolution of ambiguities, Paape et al. (2020) provide two other possible heuristics to explain their data. Since assessing the use of heuristics was not their primary objective but rather a *post hoc* explanation for their results, I have not included that study in this section, but still believe their suggestions are worth mentioning here. They suggest negation cancellation and negate the verb as heuristics that people might employ to understand sentences. The first, negation cancellation, refers to two negatives canceling each other out, and the latter, negate the verb, refers to applying any negation in the sentence to the verb. Although they did not test these directly, they suggest other possible heuristics that may be at play.

#### 3.2.1 Interim discussion

The set of studies addressing the role of heuristics in sentence processing is quite heterogeneous. It includes a variety of methods in terms of presentation modes (auditorily, self-paced reading, and rapid visual presentation), measurements (agent/patient role identification questions, sentence completion, yes/no comprehension questions, and reading times) combining online and offline measures, and types of sentences (passives, object clefts, SO/OS sentences, subject/object relative clauses, and quantifier scope ambiguous sentences). These also test different kinds of heuristics (NVN strategy, morphological case marking, frequency distribution of syntactic structures, and lexical-pragmatic associations of words) as well as different languages (English, German, and Hebrew).

As advanced already, part of this variability is due to a lack of specificity concerning which heuristics might be at play. This has also led to mixed results. Participants are better at understanding sentences where the order is subject-verb-object than when it is inverted, regardless of the frequency of that structure in the language. Order inversion is especially a problem when the sentence is not plausible, with plausibility predicting performance in various reading tasks. Additionally, higher likelihood structures are given preference (i.e., considered more likely) in comprehension over lower likelihood structures, although this is not enough to explain the NVN strategy or account for its effects. With respect to the lexical-pragmatic associations of words, participants took longer to read the dispreferred single continuations regardless of ambiguity, suggesting that pragmatic preference takes precedence over ambiguity in sentence comprehension. Nevertheless, morphological case marking was not used by participants to aid their comprehension of sentences. This result raises several questions. First, can using morphological case marking be considered a heuristic or is it part of syntactic processing? Second, even if morphological processing is not being used as a heuristic, is it possible that other heuristics are being utilized? Finally, it can be argued that there might be a hierarchy within heuristics and the NVN strategy is simply too salient or dominant to show morphological case effects (as word order was also manipulated).

In sum, it seems like there is some evidence for an NVN strategy and the use of prior information such as world knowledge and knowledge of structure frequency or likelihood. But, other heuristics may be also at play, as suggested above. It is clear that we still do not have a clear grasp of exactly what shortcuts people use when comprehending sentences. More work should be done on defining which heuristics we use and how the heuristic and algorithmic analyses relate to each other—i.e., are they sequentially done or do they occur simultaneously? In any case, a more definite demarcation of the extents and limitations of heuristics proposed by good enough processing is necessary to provide a full picture of how processing is carried out and what the response to these questions might be.

### 3.3 Deep processing is only carried out if required by the task

Perhaps the key claim of good enough processing is that processing rises to meet the demands of the task. According to Ferreira et al. (2002), we usually interpret sentences in a superficial way unless the task at hand requires deeper processing. In other words, processing is shallow unless the task demands deeper processing, in which case processing becomes more elaborate. This concept is strongly connected to the idea of heuristics and of maintaining ambiguities, as shallow representations are the consequence of a “quick and dirty” analysis carried out using rules-of-thumb and may result in keeping ambiguities unresolved. Although it is not clear whether deep and shallow processing is binary or linear and whether this idea applies to processing overall or only to syntax, several studies tested this idea of task-dependent processing. In the following paragraphs, I summarize the eight studies that address this claim directly. The evidence for this claim will be presented roughly divided into evidence of shallow processing and evidence of task-related depth of processing.

Newman et al. (2012) tested whether semantic relatedness modulated depth of processing. They looked at whether English speakers’ ability to detect morphosyntactic (subject-verb agreement) violations was affected by whether the constituents of the sentence were semantically related. They created sentences that violated subject-verb agreement in which the noun and verb were either related or unrelated.

No violation.

Related: *The bellman **serves** the traveler and carried the bags to the room.*

Unrelated: *The boy **finds** the mayor and pokes the mouse with a stick*.

Agreement violation.

Related: *The bellman **serve** the traveler and carried the bags to the room.*

Unrelated: *The boy **find** the mayor and pokes the mouse with a stick*.

They assumed that if processing is shallow or good enough, then semantic relatedness would increase the likelihood of missing a morphosyntactic violation. The idea is that if the input is plausible, then there is less of a need for a detailed analysis. They carried out both an fMRI and a self-paced reading experiment each including acceptability judgments after every sentence. They found increased activation of the left inferior frontal gyrus for anomalous-related compared to unrelated conditions. They interpreted this as participants having to suppress the shallow interpretation given by semantics to answer questions that required access to the syntax. Behaviorally, in anomalous sentences, relatedness elicited more errors and slower reading and response times. Both behaviorally and neurologically, the results inverted for the non-anomalous sentences—i.e., they showed opposite activation patterns for related and unrelated sentences compared to the anomalous sentences and improved performance for related sentences. The authors interpreted these results in support of good enough processing. They claim that we carry out shallow processing in instances when the content of the input is plausible or familiar, supporting the good enough processing claim that depth of processing depends on task requirements.

Sanford et al. (2011) tested whether semantic fit modulated depth of processing. Manipulating semantic relatedness of the anomalous word in a sentence, they tested English speakers on anomaly detection. They used ERPs to test the online processing of anomalies and asked participants whether they detected an anomaly or not. Their main manipulation was whether anomalies were easy-to-detect—poor-fit anomalies where the changed word did not fit the context—or difficult-to-detect anomalies—anomalies in which the changed word fit the general context well. An example of the first case would be:

Easy to detect or poor-fit anomaly: *Yesterday, the record shop owner told him that he would have to think of new ways to sell more **letters**/records.*

Difficult to detect anomaly: *In a recent trial, a 10-year sentence/care order was given to the **victim***.

Note that, in both cases, the bolded word marks the anomaly. They found that easy-to-detect anomalies produced an N400—highlighting a lack of goodness-of-fit—, whereas the difficult-to-detect ones did not. Instead the latter only showed a late posterior positivity around 800 ms (present in both types of anomalous stimuli)—showing the use of explicit judgment or analysis. The authors interpret these results—in particular, the differential neural signal for easy- and hard-to-detect anomalies—as evidence of differences in the processing of the two. They find, much like Newman et al. (2012), that when semantic information fits the situation well, then processing remains shallow making anomalies more difficult to detect, supporting Ferreira et al.’s claim.

Sturt et al. (2004) tested the depth of semantic processing during reading in English. They tested whether relatedness and focus affected the depth of processing. In a change-detection paradigm, participants were asked to detect words that changed in one of two focus conditions and two relatedness conditions. Focus was manipulated in one of two ways: using it-cleft sentences (emphasis on *Jamie*: *It was Jamie who really liked the cider, apparently*) and pseudocleft sentences (emphasis on *the cider*: *What Jamie really liked was the cider, apparently*) (Experiment 1) or using a context sentence (Experiment 2). Relatedness was manipulated within the word that changed (related: *cider* to *beer*; and unrelated: *cider* to *music*).

Experiment 1.

Emphasis on the subject of the action (it-cleft): It was Jamie who really liked the *cider*, apparently.

Emphasis on the object (pseudocleft): What Jamie really liked was the *cider*, apparently.

Experiment 2.

Focused context: Everybody was wondering which man got into trouble.

Unfocused context: Everybody was wondering what was going on that night.

Target sentence: In fact, the man with the *hat* was arrested.

[*Note:* The italicized word is the one that changed].

They found that if the changed word was unfocused and had changed between related words, they were more difficult to detect than when it was focused and changed between unrelated words. The authors interpreted these results as showing that participants’ representations are only good enough. They maintain that the analysis of the sentence is shallow, putting more focus on the general meaning than the specifics of the lexical item unless there is a marker calling attention to the specific word. From my point of view, this also suggests that not all parts of a sentence are processed equally well, suggesting that depth of processing is not a binary (yes or no) sentence-wide decision.

Dwivedi (2013)—explained already—tested the task-dependent depth of processing in the second and third experiments of their study. She tested whether the type of question asked after reading affected the depth of processing. She tested participants using sentences with quantifier scope ambiguity (e.g., *Every boy climbed a tree*) and continuation sentences that disambiguated number. All context sentences were biased towards a plural interpretation (e.g., *Every boy climbed a tree* tends to be interpreted as there being several trees), and the continuation sentence either matched or did not match this number preference. These were followed either by no questions or comprehension questions about number (e.g., *How many trees were climbed?*) in Experiments 1 and 2, respectively. The author found no difference in reading times between conditions in two experiments with the same manipulation. In a third experiment, she used sentences that did not show a preference for one or the other interpretation. In all cases, the reading times did not show an effect of continuation. The author did find that the effect of the dispreferred single interpretation (as measured by a norming study) in Experiments 1 and 2 depended on the task that came after. If the task did not demand deeper processing—as in Experiment 1—participants processed sentences in a shallow manner, showing no effect of mismatch between the preferred interpretation of the quantifier scope ambiguity for that stimulus and the continuation sentence. If deeper processing was required by the task as in the case of the number question of Experiment 2, then participants showed an effect of preference mismatch and thus deeper processing. The author interpreted these results as supporting good enough processing and showing a heuristic first, algorithmic second processing strategy in which the second stage is only carried out if required by the task.

Kharkwal and Stromswold (2014) tested the effects of task or stimulus variability on depth of processing. They tested whether including different types of sentences—and thus requiring more effort for comprehension—affected the depth of processing. They showed native English speakers videos of two objects (one leading and the other following) with one-sentence descriptions of the scene (e.g., *the triangle is following the circle*). Participants were asked simply to indicate whether the sentence matched the scene or not. Their accuracy and response times were measured. In one experiment, participants only saw active sentences whereas in the second they also read passives. There were two additional linguistic manipulations: the perspective of the verb—namely, source-to-goal verbs such as *chase* or *follow* or goal-to-source verbs such as *flee* or *lead*—and the verb choice (*chase* and *flee* versus *lead* and *follow*). In the first experiment, the authors found no effect of the linguistic manipulations, whereas in the second they did as well as a general slowing down in response times for *follow* and *trail* relative to *lead* and *guide*. They attributed this difference to a more detailed syntactic representation in Experiment 2 brought about by the use of passives, which led to additional costs of verifying the sentence. In other words, the stimulus context made the task more difficult, thus requiring deeper processing to be carried out, leaving room for more subtle effects to arise. The authors interpreted this as support for the claim from good enough processing that task-related demands can lead to deeper processing.

Swets et al. (2008) tested whether goal or task can influence parsing strategies—i.e., shallow versus deep. As mentioned before, they showed participants ambiguous sentences and asked them either superficial or more complex questions specifically about the relative clause. They found an ambiguity advantage in reading times—as has been observed before—, but only when participants expected superficial comprehension questions. This suggests that their analysis of sentences was dependent on the task—in this case, the type of question—with shallow questions leading to shallower processing that allows for ambiguities to be left unresolved but deeper processing showing no such allowance. Similarly, in their replication of this study with L2 speakers of English, Tan and Foltz (2020) found that the question they asked (namely, relative clause questions such as *Did the maid/princess/son scratch in public?* Or comprehension questions such as *Was anyone humiliated/proud?*) modulated reading times and that reading times to ambiguous sentences were faster than those of unambiguous sentences. The authors of both of these studies interpreted their results in support of task-dependent processing as good enough processing suggests.

Gilbert et al. (2021) tested whether the task affected the depth of processing. They tested whether changing the task would lead participants to either reanalyze the sentence or omit reanalysis. As mentioned above, they primed the less frequent meaning of polysemous target words. They found that priming for post-target disambiguated sentences depended on the task. In other words, the type of processing required by the task affected the results, with priming occurring only when deeper processing was required. They interpreted this as evidence that task requirements affect whether we take note of the specific word that was primed, as Ferreira et al. (2002) suggest.

Bader and Meng (2018) tested whether task affects misinterpretation effects by evoking deeper processing. They replicated Bader and Meng (2018)—described above—but introduced a different task. They tested native German speakers’ contrasting processing of active sentences with SO order and both OS order and passive sentences. The latter two groups were composed of non-reversible sentences—meaning that if the agent and patient roles were reversed, the sentence no longer made sense. They asked participants both plausibility and agent/patient questions for each item, in different orders by experiment. They found that accuracy for agent/patient questions was lower for OS sentences than SO and passive sentences, even in cases with correct plausibility judgments. Given that the plausibility judgments still required correct thematic role assignment—particularly in the case of non-reversible sentences—and that both tasks were carried out in close succession for each trial, the authors suggest that there cannot be a difference in depth of processing between a thematic role assignment task and the plausibility task. The authors suggest that the thematic role assignment and plausibility results should be aligned to support good enough processing. The authors propose instead that participants can produce a plausibility response immediately upon reading the sentence whereas they depend on the agent/patient probe to respond to that task. They suggest that this delay allows for memory decay and errors in retrieval, rather than the difference in processing that good enough processing would predict.

#### 3.3.1 Interim discussion

The studies that have tested depth of processing have focused on quite different measures and types of stimuli, although almost exclusively on English (with one exception looking at German). They used both online (BOLD response, ERPs, and self-paced reading times) and offline measures (accuracy and response time to various types of questions). They used very different sentences including morphosyntactic violations, subject-verb agreement violations, it-cleft and pseudocleft structures, quantifier scope ambiguity, descriptions of scenes, goal-to-source and source-to-goal verbs, and non-reversible SO and OS active and passive sentences. They also looked at various ways of manipulating task demands or inducing deeper processing including semantic relatedness, semantic fit, focus, type of question (general comprehension question versus about number or complex questions about the relative clause, or agent/patient questions versus plausibility judgments), and type of stimuli included in the task (e.g., both actives and passives versus an actives-only task).

What these papers as a whole show is fairly good evidence for shallow processing, with the important caveat that there are circumstances that cue deeper processing, such as focus or different tasks. From these studies, we can deduce that semantic relatedness and fit, focus, type of question, and context all affect depth of processing. Relatedness increases the likelihood of missing a morphosyntactic violation, poor semantic fit leads to different—in theory, shallower—kinds of processing as shown by ERP response patterns, focus leads to deeper processing, general comprehension questions lead to shallower processing than relative clause questions, and variability in the stimuli and context make the task more difficult and requires deeper processing. But, it is still unclear how or why some questions would lead to a differential depth of processing. For example, when plausibility and thematic role assignment both should in theory require the same depth of processing, the effects do not always align—i.e., participants make more mistakes in the former than the latter. In other words, these studies show some evidence that the depth of processing of a sentence is modulated by the difficulty of the task, but this evidence is not unequivocal. It is clear that questions about the syntactic structure and/or the roles of the different noun phrases within the sentence increase the depth of processing, but it is unclear whether this is always the case online or whether it affects later reanalysis. Furthermore, it is not clear precisely what kinds of questions elicit deeper processing and whether this is a gradient or a heuristic/shallow versus algorithmic/deep dichotomous kind of processing.

## 4 Discussion

In the 20 years since its original publication, the paper by Ferreira et al. (2002)—as well as its core idea of good enough processing—has gained significant traction. In the current review, I summarize the articles published during that time that explicitly address the main claims of good enough processing, posited in Ferreira et al. (2002). These claims are that when we process sentences we often leave ambiguities unresolved, use heuristics, and engage in shallow processing, proceeding to deeper processing only if the task at hand requires it. These studies present evidence from various techniques—eye tracking, EEG, fMRI, and online and offline behavioral measures—, various types of stimuli—garden-path sentences; passive, active, subject cleft, and object cleft sentences; quantifier ambiguous sentences; and subject and object relative clauses—, and various languages—English, Hebrew, German, Japanese, Spanish, and Dutch. This strengthens some claims but provides mixed evidence for others. In particular, ambiguities remaining unresolved and processing being task dependent are the two claims that have received the most direct attention and have the strongest evidence. The claim that remains the most unclear and with the most mixed evidence is our use of heuristics. While there seems to be clear support for our use of the NVN strategy, it is unclear what other heuristics we might be employing and whether, for example, the use of prior knowledge or world knowledge to assess the acceptability of a sentence can be considered a heuristic.

Furthermore, the ideas put forth by these claims are closely interrelated. Shallow processing is aided by heuristics and allows for ambiguities to remain unresolved, while when this processing is insufficient for the present purposes, deeper processing is carried out. Importantly, it is unclear whether this explanation is better than other alternatives. As a reviewer pointed out, other views of language processing provide different accounts of these phenomena. Bayesian models (e.g., Norris and McQueen, 2008) explain these in terms of interpretations being based on priors, probability distributions (analogous to heuristics presented here), and reassessments of these probabilities as later information affects the probability of an earlier segment (similar to claims or maintenance of ambiguity). Others have provided evidence that the detail of the signal is preserved for later reevaluation as opposed to maintaining ambuguity (Gwilliams et al., 2018) and the use of probabilistic speech cues as prior uncertainty is manipulated (Clayards et al., 2008). Although some of these do not test sentence processing, the same principles could also be applied. Additionally, the issue of maintaining ambiguity and garden-path sentences has also been addressed in the serial (e.g., Frazier and Fodor, 1978; Frazier and Rayner, 1982) versus parallel processing discussion (e.g., McClelland et al., 1989). Nevertheless, the focus of the current review remains good enough processing. Finally, although these claims have received a lot of attention, there are many issues that remain unresolved by good enough processing.

Looking superficially at the number of citations for Ferreira et al. (2002)—over 500—one could easily conclude that there is significant support for good enough processing. The first question to ask ourselves is what do the data actually say? Unfortunately, the answer is that the evidence is limited and unclear. When looking into the citations in more detail, it is clear that the overwhelming majority of papers reference this idea to contextualize their study (~300) or use it as a *post hoc* explanation for unexpected results (~200). This tells us two things: good enough processing has substantial intuitive appeal and a wide array of applications.

Nevertheless, when looking at the relatively few studies that attempt to test this good enough processing directly (little over 20 out of the 500+ citations), we are faced with unavoidable issues. Different authors take different interpretations of it and its predictions making it unclear whether they are truly testing it or not. We can take the idea of unresolved ambiguity as an example. For one, several studies test only temporary ambiguity. Gilbert et al. (2021) define polysemous words as ambiguous, even though they are clarified by the context and other studies consider garden-path sentences ambiguous even though they are clear by the end of the statement (e.g., Nakamura and Arai, 2016). Others consider only ambiguities that cannot be resolved within the sentence, syntactically (e.g., Swets et al., 2008; Tan and Foltz, 2020) or semantically (e.g., Dwivedi, 2013). This also relates to an underspecification of the theory in terms of whether it addresses semantic or syntactic processing, or perhaps both. Beyond the consideration of ambiguity, there are cases in which the results of a study are interpreted as evidence against the theory but could easily be taken as evidence for it following another definition. For example, Chromy (2022) concludes that garden-path sentences are just difficult to parse—rather than maintaining ambiguity. On the other hand, if we consider that grammatical sentences with only one correct syntactical analysis are sometimes treated as ambiguous (Keshev and Meltzer-Asscher, 2021), one could simply conclude that they are maintained ambiguous even after the disambiguation point. These conflicts point to a major limitation; namely, that, as it stands, good enough processing is too vague to be falsifiable. This means that, as I have pointed out at certain points, most reasonable results could be explained within this theory. Although this might seem as strong support for good enough processing, in fact, if all possible results can be interpreted as support for a theory, then the theory loses predictive value and usefulness. For example, if participants show deeper processing, given the vagueness of the definition of shallow and deep processing and what necessitates it, it would be easy to argue that the task simply requires it, even if *a priori* the assumption was that it did not.

The main reason why these issues arise, from my perspective, is that what Ferreira and colleagues propose is not a theory *per se*, but rather a guiding principle. In fact, the authors in the original and subsequent papers (Ferreira et al., 2002, 2009; Ferreira and Patson, 2007; Ferreira and Lowder, 2016; Goldberg and Ferreira, 2022) refer to the ideas of good enough representations, good enough approach, good enough processing, and the notion of good enough production/comprehension, but do not state or claim a theory exactly. Yet, others have referred to it as a theory (Logačev and Vasishth, 2016; Christianson et al., 2017, 2023; Zhou et al., 2018; Chmiel et al., 2020; Huettig et al., 2020; Karimi and Diaz, 2021; Lopukhina et al., 2022). In other words, there is no presentation of “this is how people process language step-by-step,” but rather a rejection of the idea that we carry out complete, “perfect” processing of language in everyday situations. This draws attention to the goals of comprehenders who are not linguists explicitly analyzing a sentence. They argue that rather than focusing on accuracy and detail, the comprehension system’s goal is to allow the listener to formulate an appropriate response to the speaker—be it an oral response, an action, or even a gesture of agreement. This rings particularly true for conversation and natural speech. It is reasonable and even beneficial if we consider speech as portrayed above—noisy and error-prone. Good enough processing points to strategies for dealing with this, while keeping in mind limited resources and the natural tendency to economize them. This is in stark contrast with the idea of a full syntactic tree-like interpretation or parsing of a sentence that dominates theories of comprehension.

The issue remains, though, that for each of the claims of good enough processing, the mechanisms and specifics are unclear. For example, are these claims about semantic processing, syntactic processing, or both? Concerning maintaining ambiguities, it is unclear exactly how this is achieved. For this reason, varying interpretations and predictions arise, highlighting this limitation that does not allow good enough processing to be a full theory. For one, maintaining multiple representations in mind should show opposite effects to those presented here, requiring more working memory rather than less. But, good enough processing does not provide a clear explanation of how it is that maintaining multiple representations is more efficient in the short term. With respect to the second claim and as suggested above, it is important to clarify exactly which heuristics come into play as well as how these shape comprehension. For example, do they guide comprehension only when there is ambiguity or do they provide blueprints, so to speak, that always guide our understanding? Concerning shallow and deeper processing, we need to characterize these phenomena better. Is the depth of processing dichotomous, being either algorithmic or heuristic or are there various levels? Does one kind of processing occur first and then the other or are they simultaneous or continuous? There have been various attempts at this last question, with some suggesting the shallow is followed by a deep processing and others suggesting a simultaneous start approach. This is an essential issue that leaves many questions unanswered. For example, what is the stopping rule or how do we determine the kind of processing that is necessary or good enough? Furthermore, if the depth of processing is a spectrum—either linear or multilayered—is this one continuum between shallow and deep (meaning if you require deep processing of the lexical items, you will process the grammar deeply too and vice versa—also begging the question of how shallow is shallow and how deep is deep) or is it a combination of category dependent choices—e.g., deep processing of the lexical items but shallow grammatical processing?

Although this idea of good enough processing is quite appealing, the question of what we actually carry out is not clear. I believe that the explanatory power and common-sense appeal are at the heart of both the attraction and innovativeness of good enough processing. It should be highlighted that multiple theories claim multiple layers or stages of processing which brings up the question of what differentiates this theory from those. Perhaps the nature of these stages is unique, but this is not clear from the proposition as it stands. Clearly, the traction this idea has gained makes a strong suggestion that something *must* be different or unique about it. One could argue that what differentiates it from others is that it claims that deeper processing (or the subsequent layers of processing) is not mandatory and that common processing is shallow or “good enough.” One possible corollary of good enough processing is a claim that we never carry out fine-grained analyses. But, as the authors correctly point out, we can carry out these analyses when the task requires it. What is unclear is what exactly determines whether a task requires deeper processing and how we—as language processors—know or determine whether shallow processing suffices or if deeper processing is required.

## 5 Conclusion

Although good enough processing has attracted a lot of attention and has been cited amply, the evidence for it is quite mixed. This suggests that it provides a very promising perspective, but much work needs to be done to assert it as a theory. As it stands, much of the content of good enough processing relates to various processes that may be at play and relies more on providing a characterization of phenomena than on providing a mechanistic account of comprehension. In other words, it does not make direct predictions but rather provides a possible logic of the system. For this to provide an explanatory and predictive account, we must clarify exactly the scope and limitations of each of these claims as well as assess the possible mechanisms for each.

There is much work to be done, but it is clear that the idea of good enough processing has gathered some well-earned attention and traction over the years. The seminal 2002 paper has given us an alternative theoretical approach to sentence comprehension, opening up both many questions and many promising answers. Thanks to this, we have formulated nuanced alternatives to deterministic algorithmic processing of language and are hopefully closer to understanding how this complex system of language functions.

## Author contributions

CF: Conceptualization, Methodology, Project administration, Writing – original draft, Writing – review & editing.
